# The Power of Metabolism for Predicting Microbial Community Dynamics

**DOI:** 10.1128/mSystems.00146-19

**Published:** 2019-06-11

**Authors:** Jeremy M. Chacón, William R. Harcombe

**Affiliations:** a Ecology, Evolution, and Behavior, University of Minnesota, St. Paul, Minnesota, USA; b Biotechnology Institute, University of Minnesota, St. Paul, Minnesota, USA

**Keywords:** metabolism, antibiotics, bacteriophage, ecology, evolution, genome-scale modeling, systems biology

## Abstract

Quantitative understanding and prediction of microbial community dynamics are an outstanding challenge. We test the hypothesis that metabolic mechanisms provide a foundation for accurate prediction of dynamics in microbial systems. In our research, metabolic models have been able to accurately predict species interactions, evolutionary trajectories, and response to perturbation in simple synthetic consortia. However, metabolic models have many constraints and often serve best as null models to identify additional processes at play. We anticipate that major advances in metabolic systems biology will involve scaling bottom-up approaches to complex communities and expanding the processes that are incorporated in a metabolic perspective. Ultimately, cellular metabolism will inform predictive ecology that enables precision management of microbial systems.

## Abstract (precis)

Quantitative understanding and prediction of microbial community dynamics are an outstanding challenge. We test the hypothesis that metabolic mechanisms provide a foundation for accurate prediction of dynamics in microbial systems. In our research, metabolic models have been able to accurately predict species interactions, evolutionary trajectories, and response to perturbation in simple synthetic consortia.

## PERSPECTIVE

Precise prediction and management of microbial community dynamics are a goal of personalized medicine, industrial biotechnology, and environmental management. A fundamental aspect of this challenge is understanding how community composition and function emerge from lower-level information like species' metabolism. Metabolism determines the nutrients that a cell needs to survive and reproduce as well as the compounds that a cell excretes into its environment. This basic information leads to the hypothesis that an understanding of metabolism and the nutrient environment can allow for prediction of bacterial phenotypes, species interactions, and emergent community properties. Since modern genomics and systems biology have provided us with the ability to predict species’ metabolisms from sequence data, we can now rigorously test this hypothesis. In this Perspective, we will use examples from our research to argue that understanding species’ metabolism does in fact allow us to accurately predict microbial interactions, evolution, and community dynamics. However, we will also discuss important exceptions, where metabolic predictions failed. We’ll conclude by discussing what future research we think should be prioritized to reduce the limitations currently faced by metabolic systems biology.

We use several tools to quantitatively predict, and rigorously test, how microbial community dynamics emerge from intracellular metabolism. Primarily, we use dynamic genome-scale metabolic modeling to predict optimal microbial physiology from cells’ intracellular metabolic networks and the resources available in the environment (1, 2). This method quantitatively predicts the multispecies ecosystem dynamics which emerge from cells’ underlying metabolic capabilities by iterating through optimization of strain-specific models and updating the environment through time (1). We frequently supplement these genome-scale models with simpler Monod models (e.g., reference 3) to investigate general principles about how metabolites structure microbial systems, as well as to include nonmetabolic phenomena. We focus our predictions on systems comprised of some combination of the model organisms Escherichia coli, Salmonella enterica, and Methylobacterium extorquens. We engineered a strain of each species so that we can toggle interactions from obligate cross-feeding mutualism to competition simply by changing the nutrients provided. Furthermore, we have curated genome-scale metabolic models of each strain, which reduces the chance that metabolic predictions will fail due to annotation errors.

We have found that metabolism is sufficient to predict species interactions and equilibrium community composition in different nutrient environments. Genome-scale metabolic modeling was able to predict *a priori* the environment-specific interdependency of our 3-species mutualism, as well as the species ratios to which the community converged (1). Metabolic modeling was also instrumental in understanding how changes in available nutrients impact interactions and maintenance of diversity in cross-feeding systems. Models highlighted that as dependency on other species decreased, community stability depended on growth rates and which nutrients became limiting (3). Metabolism also drives the impact that spatial location has on bacterial interactions. For example, we were able to show that the rate of nutrient uptake strongly influences the scale over which colonies interact (4). The degree to which a colony competed only with neighbors increased predictably with increasing nutrient uptake rates and decreasing nutrient diffusion. The success of these simple explorations is promising for our ability to predict community dynamics. However, failures have also been informative. Quantitative divergence in the expected scale of competition allowed us to identify autoinhibition by toxic waste products (4). Incorporation of this unexpected self-poisoning as a modifier of growth metabolism was critical for understanding dynamics in the system. Toxicity as a modifier of metabolism is likely to be a common theme in microbial communities (5).

Beyond ecology, a metabolic approach has allowed us to make successful predictions about the evolutionary trajectory of metabolically interdependent species. Game theory and other approaches provide powerful theory for predicting the evolution of social interactions (6). However, this theory tends to provide qualitative predictions that rely on assumed costs and benefits. By incorporating metabolism, it is possible to quantify fitness effects based on stoichiometric tradeoffs and to predict the mechanistic basis of adaptation. For example, metabolic mechanisms were useful for understanding how adding an exploiter impacted selection for cooperation in a bipartite mutualism (7). Metabolic modeling predicted that addition of the exploiter in a structured environment would increase selection for cooperation, by increasing the variance in nutrient concentrations. Social evolutionary theory predicted the opposite: that adding an exploiter would make cooperation an unfit strategy. The metabolic models ultimately proved more accurate and helped us understand the mechanisms underlying experimentally observed evolution. Dynamic, genome-scale metabolic modeling has also proven capable of predicting the genetic basis of adaptation. We computationally predicted the knockouts that would have the greatest impact on species' fitness in a mutualism (8) and then subsequently observed that one of the most consequential knockouts repeatedly evolved in long-term experiments (9). As an important caveat, the observed mutation was one of several solutions predicted to be equally optimal. The fact that we repeatedly observed the same solution versus a mixture of equally optimal solutions suggests that some important biological constraints such as gene regulation were missing from the model.

Metabolism has also proven to be surprisingly useful for predicting how communities will respond to nonmetabolic perturbations. A metabolic approach predicts that different types of metabolic interactions should generate different types of response to a given perturbation. If species are engaged in nutrient competition, then inhibition of a single species tends to increase the abundance of competitors, altering species ratios but having little impact on total biomass (8). In contrast, metabolic interdependencies tend to constrain species ratios such that inhibition of any species reduces the abundance of all interdependent members of the community. In one experiment, we challenged our three-species synthetic community with antibiotics in environments that either caused competition or required interdependency (10). When the species were competing for metabolites, ecosystem productivity was not greatly affected until the antibiotic concentration was high enough to kill all species. When the species were metabolically interdependent, however, the growth of all species was limited by the most drug-sensitive member (the “weakest link”). This growth limitation also occurred when we challenged an E. coli/S. enterica interdependency with an S. enterica bacteriophage. However, initial metabolic predictions were not supported when we used a phage that killed E. coli. Instead, we saw an increase in abundance of metabolically dependent S. enterica (11). A closer examination revealed a metabolic explanation: phage lysis released nutrients in the environment which the metabolic partner could then scavenge, reminiscent of the “viral shunt” which cycles nutrients in marine food webs (12). We did not predict this impact of phage *a priori*, though modifying our models to include the released nutrients allowed for the experimental results to be qualitatively recapitulated with a metabolic approach. On the one hand, the iteration between model and experiment has proven to be a valuable tool for reconciling deviations from metabolic theory with the underlying metabolic framework. On the other hand, such iteration is intensive and will assuredly be harder as we move to more complex communities.

Our work has found a metabolic perspective to be a successful predictor of some, but not all, dynamics in small, well-defined consortia. Importantly, even when predictions were incorrect they served as a useful null model that helped us to identify processes that could be incorporated to explain observed community dynamics. It should be noted, however, that the power to make useful predictions in our system is strongly influenced by using well-curated models in environments with defined nutrients. Looking forward, we envision two essential tasks. First, we need to improve methods for using bottom-up approaches for predicting dynamics in complex communities. In part, this will involve improving the accuracy, and therefore predictive power, of metabolic models for uncharacterized species, for example, through better methods to gap-fill and predict biomass composition (13). Additionally, we are likely to need hybrid approaches that allow metabolic prediction in the context of uncertain environments. Incorporation of phenomenological information, for example, by measuring net ecological interactions (14), may be required when detailed metabolic information is unavailable. A second major task will be to continue to enlarge our vision of metabolism. We predict that transformative research will come from developing a mechanistic understanding of how extrametabolic processes influence metabolism. We have found that incorporating nonmetabolic phenomena (e.g., phage and toxicity) in terms of their metabolic effects paved the way to accurate predictions. Optimistically, continued research may find that nonmetabolic phenomena can be boxed into a few categories of metabolic effects, perhaps such as those which alter the metabolic rates and capabilities of cells (e.g., regulation, toxins, temperature, and pH) and those whose effects alter the nutrient environments (e.g., lytic phage and hosts). Work has begun in this direction, but there is still much to be done (15). A central goal should be to determine major categories of metabolic modifiers, predict the information computationally, and test these predictions in controlled and more complex communities. In this vision, the metabolic requirements, abilities, and by-products of species are the essential heart of predictive ecology, with all other phenomena acting as modifiers.
